# An fMRI dataset of social and nonsocial reward processing in young adults

**DOI:** 10.1016/j.dib.2024.110197

**Published:** 2024-02-15

**Authors:** David V. Smith, James Wyngaarden, Cooper J. Sharp, Daniel Sazhin, Ori Zaff, Dominic Fareri, Johanna Jarcho

**Affiliations:** a Temple University, United States; b University of Pennsylvania, United States; c Adelphi University, United States

**Keywords:** Functional magnetic resonance imaging, fMRI, Substance use, Decision making, Brain, Reward sensitivity

## Abstract

Trait reward sensitivity, risk for developing substance use, and mood disorders have each been linked with altered striatal responses to reward. Moreover, striatal response to reward is sensitive to social context, such as the presence of a peer, and drugs are often sought out and consumed in social contexts or as a result of social experiences. Thus, mood disorder symptoms, striatal responses to social context and social reward may play a role in substance use. To investigate this possibility, this dataset was collected as part of a National Institute on Drug Abuse (NIDA) grant titled “Aberrant Reward Sensitivity: Mechanisms Underlying Substance Use” (R03-DA046733). The overarching goal was to characterize the associations between neural responses to social and nonsocial rewards, trait reward sensitivity, substance use, and mood disorder symptoms. After obtaining questionnaire data quantifying reward sensitivity, substance use, and other psychosocial characteristics, young adults (N=59; 14 male, 45 female; mean age: 20.89 years ± 1.75 years) completed four fMRI tasks testing different features of social and reward processing. These included: 1) a strategic reward-based decision-making task with Ultimatum and Dictator Game conditions; 2) a task where participants shared rewards or losses with peers, strangers, or non-human partners; 3) a task in which participants received well-matched social and monetary rewards and punishment; and 4) a monetary incentive delay (MID) task in which participants tried to obtain or avoid rewards and losses of different magnitude. This dataset includes sociodemographic questionnaire data, anatomical, task-based fMRI, and corresponding behavioral task-based data. We outline several opportunities for extension and reuse, including exploration of individual differences, cross-task comparisons, and representational similarity analyses.

Specifications Table

**Table:** 

Subject	Biological Sciences → Neuroscience: Cognitive
Specific subject area	Social and nonsocial reward systems
Data format	Raw; BIDS-compliant
Type of data	structural brain images (compressed NIfTI files [.nii.gz]) functional brain images (compressed NIfTI files [.nii.gz]) experimental design information (tab-separated values [.tsv]) experiment meta-data (Java Script objection notation [.json])
Data collection	We collected neuroimaging data from 59 participants using a 3T Siemens Prisma MRI scanner (Syngo MR E11) with a 20-channel head coil. Functional images were acquired using a simultaneous multislice (multi-band factor = 2) gradient echo-planar imaging (EPI) sequence [240 mm in field of view (FOV), repetition time (TR) = 1,750 ms, echo time (TE) = 29 ms, voxel size of 3.0 × 3.0 × 3.0 mm^3^, flip angle = 74°, interleaved slice acquisition, with 52 axial slices]. During imaging, participants engaged in four tasks tapping into different aspects of social and nonsocial reward processing. We also collected questionnaire data using 20 different measures. All data conform to the Brain Imaging Data Structure (BIDS) standard.
Data source location	Institution: Temple University Location: Philadelphia, Pennsylvania, USA
Data accessibility	Repository name: OpenNeuro Data identification number: https://doi.org/10.18112/openneuro.ds004920.v1.1.0 Direct URL to data: https://doi.org/10.18112/openneuro.ds004920.v1.1.0 Source code: https://github.com/DVS-Lab/ISTART-DataInBrief

## 1 Value of the Data

• These fMRI data are valuable to understand how the young adult brain responds to reward-based decision-making and feedback in social and non-social contexts.
• Each task includes well-matched contrasts to test neural response to different features of social or reward processing: 1) Ultimatum vs. Dictator Game: Strategic reward-based decision-making with and without threat of social retribution; 2) Shared Reward: Sharing monetary losses or gains with a friend, stranger, or non-human partner; 3) Social Doors: differences in response to social or monetary reward (liked by a peer/win money) and punishment (disliked by a peer/lose money); 4) Monetary Incentive Delay Task: differences in response to high or low levels of gain or loss.
• Specificity or generalizability of brain function engaged during reward processing in social and nonsocial contexts can be tested in relation to numerous sociodemographic and clinically relevant individual differences measures.
• Data can be analyzed using a whole brain, region of interest (ROI), or circuit level (PPI) approach.
• Researchers interested in brain-based reward processing, and social cognition more specifically, may benefit from this dataset through its well-matched tasks and wide range of individual difference measures.
• Expanding beyond univariate analyses, researchers could use multivariate methods, such as representational similarity analysis (RSA), to test for specificity and generalizability of processing across reward decision-making contexts (with/without threat of retribution), partners (friend, stranger, non-human), and domains (social/monetary).

## 2 Background

Prior studies have shown links between trait reward sensitivity and risk for developing substance use and mood disorders [1]. Building on these observations, neuroimaging studies have shown that substance use and mood disorders are associated with altered striatal responses to reward [2]. Yet, the striatal response to reward is also sensitive to social context, such as the presence of a peer [3]. Furthermore, drugs are often sought out and consumed in social contexts or as a result of social experiences [4]. Thus, striatal responses to social context and social reward may play a role in substance use.

To investigate this possibility, this dataset was collected as part of a NIDA grant titled “Aberrant Reward Sensitivity: Mechanisms Underlying Substance Use” (R03-DA046733). The overarching goal of this project was to characterize the associations between neural responses to social and non-social rewards, trait reward sensitivity, substance use, and other psychosocial variables [5]. To date, we have published/preprinted efforts that show links between substance use and trait reward sensitivity in tasks focused on social reward [6], social context [7], and social decision making [8]. The current data article adds to these papers/preprints by describing the full scope of the dataset and outlining possibilities for reuse.

## 3 Data Description

This dataset is available as OpenNeuro Dataset 4920 [9], and it is composed of neuroimaging data from 59 participants who completed four tasks involving social and nonsocial reward processing. The data are organized in accordance with the Brain Imaging Data Structure (BIDS) specification [10] using HeuDiConv [11]. Anatomical images (T1w) were defaced using PyDeface.

As part of the BIDS standard, all files are accompanied by .json sidecars that contain metadata regarding the file. This organization is particularly important in the case of NIFTI data (.nii.gz files) and tabular data (e.g., .tsv files).

**Table:** 

Data Files	Description
participants.tsv	Basic demographics for each participant (gender, age at time of appointment, race, and ethnicity) as well as BMI, self-reported handedness, appointment drug test, appointment breathalyzer, and medication screening results.
sub-*/sub-*_scans.tsv	Four column file that describes basic information regarding the images
sub-*/anat/sub-*_T1w.nii.gz	NIfTI format T1 weighted anatomical
sub-*/fmap/sub-*_acq-bold_magnitude*.nii.gz	Magnitude component of B0 fieldmap
sub-*/fmap/sub-*_acq-bold_phasediff.nii.gz	Phase component of B0 fieldmap
sub-*/func/sub-*_task-*_run-*_bold.nii.gz	BOLD data
sub-*/func/sub-*_task-*_run-*_sbref.nii.gz	Single-band reference image
sub-*/func/sub-*_task-*_run-*_events.tsv	Five column file describing the stimulus onset, duration, trial type, response time, and stim file. Note that one task (sharedreward) has a four column events file, only excluding stim file.

## 4 Experimental Design, Materials and Methods

### 4.1 Participants

We recruited a total of 59 participants (14 male / 45 female) to engage in the fMRI tasks for this study. Across these 59 participants, the mean age was 20.89 years (minimum: 18.19; maximum: 26.28; *SD*: 1.75 years) and was mostly white (31 white, 22 Asian, 3 Black/African American, 3 other—Two or more races). Each participant completed four neuroimaging tasks (order counterbalanced). All participants gave informed consent in accordance with the Institutional Review Board at Temple University. Participants were recruited via the Temple University Psychology and Neuroscience Department participant pool, and from the surrounding community via flyers and online advertisements. Participants were paid $25 per hour for fMRI and $15 per hour for behavioral tasks, and received bonuses based on their decisions on other neuroeconomic tasks (not reported here), resulting in a total payment of $140 to $155. In addition, participants recruited from the university pool also received research credit for their participation.

### 4.2 Questionnaires

We collected self-reported data on several different facets of behavior, health, and personality. These measures are stored in the phenotype directory in the BIDS dataset. The accompanying json sidecar file contains all of the necessary metadata to reconstruct the items in the scale and the original citation for the measure.

**Table:** 

File Name	Goal of Measure
**Reward Sensitivity**	
positive_valence_systems_survey.tsv	Measures responses to reward in domains of food, physical touch, outdoors, positive feedback, social interactions, hobbies, and goals.
sensitivity_to_punishment_sensitivity_to_reward_ questionnaire.tsv	Measures sensitivity to punishment and sensitivity to reward in individual subscales.
temporal_experience_of_pleasure.tsv	Measures anticipatory and consummatory facets of pleasure.
behavioral_inhibition_scale_behavioral_activation_scale.tsv	Assesses individual differences in the sensitivity of behavioral inhibition and behavioral activation.
**Substance Use**	
adolescent_alcohol_and_drug_involvement_scale.tsv	Screens and measures an adolescent's use of alcohol and/or drug involvement.
alcohol_use_disorder_identification_test.tsv	Assesses alcohol consumption, drinking behaviors, and alcohol-related problems.
drug_use_disorder_identification_test.tsv	Screens and measures drug intake and selected criteria for substance abuse.
**Psychosocial characteristics**	
autism_quotient.tsv	Investigates whether adults of average intelligence have symptoms of autism spectrum conditions.
becks_depression_inventory.tsv	Characterizes attitudes and symptoms of depression.
altman_self_rating_mania_scale.tsv	Assesses the presence and/or severity of manic symptoms.
seven_up_seven_down.tsv	Measures manic and depressive tendencies in two individual subscales.
rosenberg_self_esteem.tsv	Measures global self-worth through negative and positive feelings about the self.
trait_emotional_intelligence.tsv	Operationalizes and measures the Trait Emotional Intelligence Theory as applied to individuals.
interpersonal_reactivity_index.tsv	Measures dispositional empathy.
personal_norms_of_reciprocity.tsv	Measures positive reciprocity, negative reciprocity, and beliefs in reciprocity.
buss_perry_aggression_questionnaire.tsv	Measures physical aggression, verbal aggression, anger, and hostility in individuals.
social_experience_questionnaire.tsv	Measures victimization through rational and overt aggression, as well as positive peer treatment.
childhood_trauma_questionnaire_short_form.tsv	Measures an individual's experiences of child abuse and neglect.
questionnaire_of_unpredicatability_in_childhood.tsv	Intended to measure unpredictability in the social, emotional, and physical domains of a child's environment.
**Interpersonal Measures: Task-based**	
inclusion_of_other_in_the_self_computer.tsv	Measures how close the respondent feels with the computer image used in tasks.
inclusion_of_other_in_the_self_friend.tsv	Measures how close the respondent feels with the friend image used in tasks.
inclusion_of_other_in_the_self_stranger.tsv	Measures how close the respondent feels with the stranger image used in the task.

### 4.3 Experimental tasks

All tasks were completed by participants while undergoing fMRI. Task order was counterbalanced across participants.

**Fig. 1:**
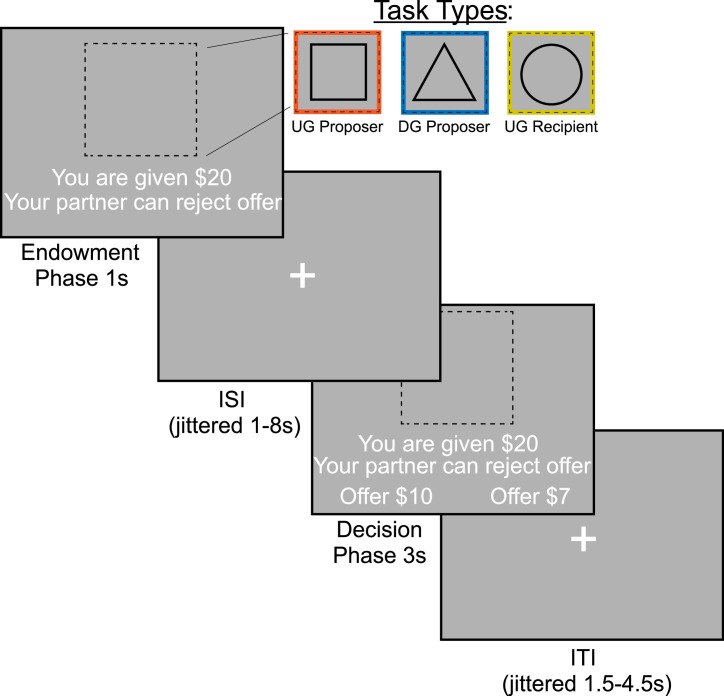
Ultimatum and Dictator Games. We operationalized strategic behavior as offering more in the Ultimatum Game and less in the Dictator Game, as this strategy would maximize earnings for the participant. During the Endowment phase, participants received $15-25. A square indicated that the participant would be acting as the Proposer in the Ultimatum Game or deciding how much money to split with a counterpart. A triangle indicated that the participant would act as the Proposer in the Dictator Game. Finally, a circle indicates that the participant would be the Recipient in the Ultimatum Game, which allowed them to decide whether they would accept or reject an offer given to them. During the Decision Phase, the participant as a proposer decided to offer More or Less to their counterpart. As a recipient, whether to Accept or Reject the offer. This Fig. was adapted from previous work with permission [8].

**Ultimatum Game and Dictator Game.** To measure strategic behavior in social contexts, we used the Dictator Game (DG; [12]) and Ultimatum Game (UG; [13]). In both games (see Fig. 1), a sum of money is divided between a proposer and a recipient. In the DG, the proposer allocates money to a passive recipient. To maximize earnings, proposers should choose to keep a greater allocation for themselves. In the UG, a recipient can reject the proposer's allocation and both players receive nothing. To maximize earnings, proposers should offer closer to an even split to avoid rejection by the recipient.

Participants made decisions in the Dictator Game as a Proposer (DG-P), Ultimatum Game as a Proposer (UG-P) and as an Ultimatum Game Recipient (UG-R). They were led to believe that each trial was played with a different participant (i.e., one shot games), proposals they received were from past participants, their own proposals would be used with future participants, and a bonus payment would be made depending on the outcome of a randomly selected proposal. We presented 72 total trials, 24 in each of the three conditions (DG-P, UG-P, UG-R), across two 7:30 minute runs (total time of ∼15 minutes). Each trial consisted of an endowment phase (1 seconds), interstimulus interval (ISI) of 1.5-4.5 seconds M = 2.42 seconds, decision phase (3 seconds), and an intertrial interval (ITI) of 1-8 seconds; M = 2.7 seconds. Each participant was endowed with a sum of money between $15–$25. To indicate whether the participant was playing as a DG-P, UG-P, or UG-R, they were presented with a target stimulus of a triangle, square, or circle respectively. During the decision phase, proposers were presented with the option to offer combinations of 6, 19, 32, or 45 percent of the endowment. Participants were able to select between two options, to offer More or Less, or Accept or Reject their offer. These options switched sides between trials. If a participant missed a trial, the screen indicated they were too slow and logged a missed trial.

**Fig. 2:**
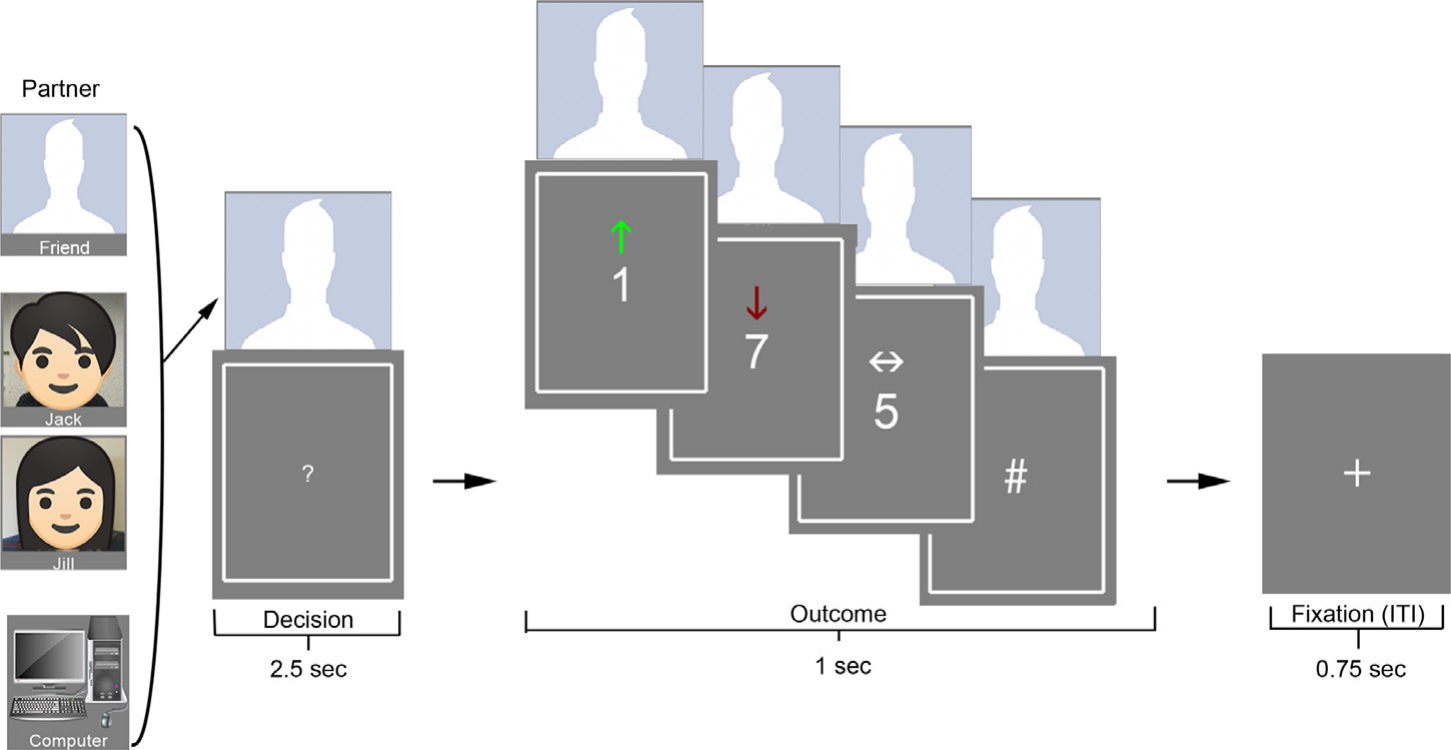
Shared Reward Task. The task consisted of a card guessing game (Delgado et al., 2000; Fareri et al., 2012) with social context manipulated through a computer, a gender-matched confederate (Jack/Jill), or a close friend condition. Participants were told that wins ($10) or losses ($5) would be shared equally with their partner. A green up-arrow indicated a correct guess and a monetary gain of $10.00; a red down-arrow indicated an incorrect guess and a monetary loss of $5.00; a white arrow pointing side-to-side indicated a neutral outcome with no money won or lost. Players guessed whether the number on a card would be above or below 5, with choices between 1 and 9. A picture of the partner's face remained above the card from the onset of decision-making through the end of the outcome phase. We measured the responses associated with winning or losing money contingent on the social context of partner type. This Fig. was adapted from previous work with permission [7].

**Shared Reward Task.** In the card guessing game (Fig. 2) [14,15], participants used their right hand's index finger or middle finger to guess whether the value of a playing card was ‘lower’ or ‘higher’ than 5, respectively. Participants were told that they would be splitting monetary gains of $10 or losses $5 evenly with one of three partners. The possible partners were: 1) a gender-matched close friend who participants put research staff in contact with; 2) a gender-matched confederate, said to be a past participant in the study, serving the role of a stranger; and 3) a non-human control in the form of a computer. Money shared with the partner on the selected trial was said to be sent to the friend or stranger via Amazon gift card, or returned to a pool of lab funds for the computer. The image of the partner for any given round appeared at the beginning of the trial phase and remained until the end of the outcome phase. Participants cycled through each partner for blocks of 8 trials, with each block primarily geared towards wins or losses (6 trials in the condition of interest, and 2 neutral or opposing conditions; cf. [16]). Two runs were completed, each lasting 6 minutes and 54 seconds.

**Fig. 3:**
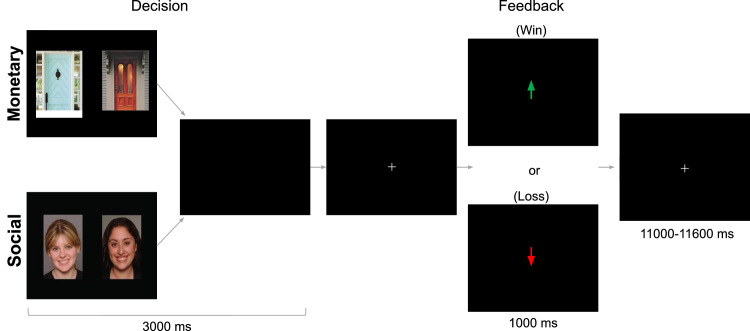
Social Doors Task. On each trial, participants choose either between two doors (monetary condition) or the faces of two peers (social condition) in search of a reward. After a brief interval, they receive feedback: an upward arrow indicating a win (monetary condition=$0.50 gain; social condition=positive peer feedback) or a downward arrow indicating a loss (monetary condition=$0.25 loss; social condition=negative peer feedback). This Fig. was adapted from previous work with permission [6].

**Social Doors Task.** The monetary and social reward task [17] were presented in separate runs, order counterbalanced across participants). This design eliminates task switching costs while still preserving our ability to directly contrast responses associated with social and nonsocial reward. In the monetary condition, participants were shown two doors. Participants were instructed to pick the door that contained a $0.50 prize (3 seconds; Fig. 3). On monetary reward trials, feedback (1 second) indicated that the participant won $0.50. On monetary loss trials, feedback indicated that the participant lost $0.25. In the social condition, participants were shown the faces of two peers. Participants were informed that each peer had indicated whether they liked or disliked the participant based on a photo of them, and they were instructed to pick the peer that liked them. On social reward trials, feedback indicated that the peer liked them. On social loss trials, feedback indicated that the peer disliked them. Each run included 60 trials: 50% resulted in reward and 50% resulted in loss feedback. Although both tasks involve feedback about correct/incorrect guesses, the result of that feedback occurs in social or monetary domains. Trials were separated by a variable duration intertrial interval (1.1 - 11.6 seconds; M = 3.5 seconds).

**Fig. 4:**
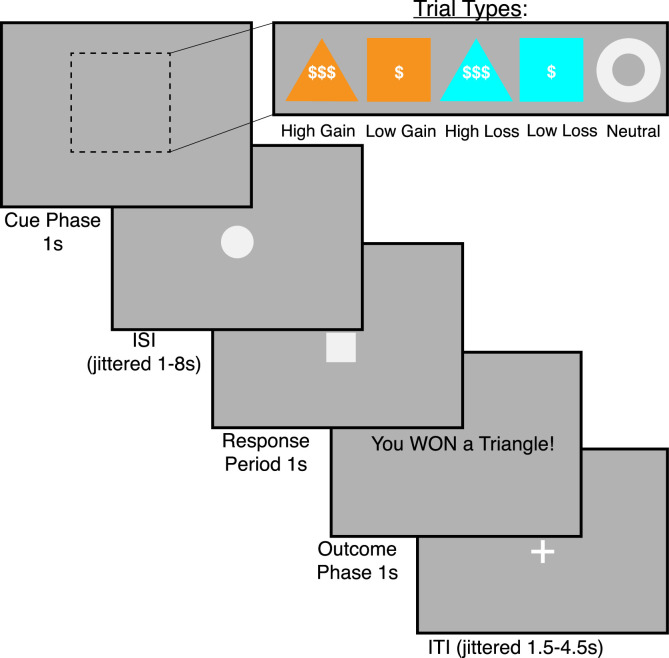
Monetary Incentive Delay Task. In the monetary incentive delay task, participants have a limited time to respond to visual cues indicating five different conditions: high gain, low gain, high loss, low loss, and neutral. Reaction times reflect the motivational impacts of the conditions. This Fig. was adapted from previous work with permission [5].

**Monetary Incentive Delay Task.** The monetary incentive delay (MID) task (e.g., [18]; Fig. 4) is comprised of five conditions in which participants respond to visual cues indicating potential monetary outcomes: high gain (orange triangle), low gain (orange square), high loss (blue triangle), low loss (blue square), and neutral (no gain or loss; white circle). During the cue phase participants are shown the condition for the current trial via presentation of one of the unique cues. This phase is followed by a jittered inter-stimulus interval, lasting from 1 to 8 seconds. At the end of the interval, a small square appears indicating the onset of the response period, where participants have 1 second to make a button press. If they respond within the response period, they win the monetary prize (gain trials) or avoid the monetary loss (loss trials). If they are too slow, they do not win the monetary prize (gain trials) or fail to avoid the monetary loss (loss trials). Reaction time serves as a proxy for motivational impact of these distinct incentive conditions.

### 4.4 MRI data acquisition

Functional images were acquired using a 3.0 Tesla Siemens PRISMA MRI scanner and a 20-channel head coil. Blood Oxygenation Level-Dependent (BOLD) sensitive functional images were acquired using a simultaneous multislice (multi-band factor = 2) gradient echo-planar imaging (EPI) sequence (240 mm in FOV, TR = 1,750 ms, TE = 29 ms, voxel size of 3.0 × 3.0 × 3.0 mm^3^, flip angle = 74°, interleaved slice acquisition, with 52 axial slices). We also collected single-band reference images with each functional run of multi-band data to improve motion correction and registration. To facilitate anatomical localization and co-registration of functional data, a high-resolution structural scan was acquired (sagittal plane) with a T1-weighted magnetization-prepared rapid acquisition gradient echo (MPRAGE) sequence (224 mm in FOV, TR = 2,400 ms, TE = 2.17 ms, voxel size of 1.0 × 1.0 × 1.0 mm^3^, flip angle 8°). In addition, we also collected a B0 fieldmap to unwarp and undistort functional images (TR: 645 ms; TE1: 4.92 ms; TE2: 7.38 ms; matrix 74 × 74; voxel size: 2.97 × 2.97 × 2.80 mm; 58 slices, with 15% gap; flip angle: 60°).

## Limitations

Our dataset has some notable limitations. First, we collected 10 scans before the COVID-19 pandemic halted data collection for 15 months. Upon resuming the study, we dropped some components of the original study due to budgetary constraints. These additional measures included diagnostic interviews that assess history of substance use and mood disorders. Second, due to an experimenter error, we are missing Inclusion of Other in Self data for the stranger condition for several participants (missing for 23/59 participants who have brain data). This omission limits some analyses that seek to compare relative closeness between friends and strangers. Third, although our sample size is larger than many neuroimaging studies [19], it may still be too small to detect some brain-behavior associations [20].

## Ethics Statement

All participants provided written informed consent to engage in the study and share their de-identified data publicly. The study was approved by the Institutional Review Board at Temple University (Philadelphia, Pennsylvania, USA) under Protocol Number 24452, and it was conducted in accordance with the Declaration of Helsinki.

## CRediT authorship contribution statement

**David V. Smith:** Conceptualization, Methodology, Software, Formal analysis, Data curation, Writing – original draft, Writing – review & editing, Supervision, Project administration, Funding acquisition. **James Wyngaarden:** Methodology, Software, Formal analysis, Investigation, Data curation, Writing – original draft, Writing – review & editing, Visualization. **Cooper J. Sharp:** Methodology, Software, Formal analysis, Investigation, Data curation, Writing – original draft, Writing – review & editing. **Daniel Sazhin:** Methodology, Software, Formal analysis, Investigation, Data curation, Writing – original draft, Writing – review & editing, Visualization. **Ori Zaff:** Methodology, Software, Formal analysis, Investigation, Data curation, Writing – original draft, Writing – review & editing, Visualization. **Dominic Fareri:** Conceptualization, Methodology, Software, Writing – review & editing. **Johanna Jarcho:** Conceptualization, Methodology, Writing – original draft, Writing – review & editing, Supervision, Project administration, Funding acquisition.

## Data Availability

An fMRI dataset of social and nonsocial reward processing in young adults (Original data) (OpenNeuro).
